# A Rare Presentation of Stage IV Large Cell Neuroendocrine Carcinoma of the Cervix with Metastasis to the Cranium

**DOI:** 10.1155/2018/2812306

**Published:** 2018-06-14

**Authors:** Neema Hooker, Sveta Mohanan, R. Tucker Burks

**Affiliations:** Atrium Health, Family Medicine Residency, 2001 Vail Ave, Charlotte, NC 28207, USA

## Abstract

Neuroendocrine tumors (NETs) are aggressive diseases developing from neuroendocrine cells that most frequently involve the gastro-entero-pancreatic tract and the lung, but more rarely are found in almost all body tissues. Limited biological and clinical data are currently available for NETs in uncommon sites, such as female genital tract. NETs represent 0.9% to 1.5% of the tumors of the uterine cervix. This case is reported on a 75-year-old Caucasian woman, presenting with dental and generalized pain for two weeks. Later during her admission, facial droop and diplopia were noticed. Radiological findings ruled out cerebrovascular accident but revealed multiple bone marrow lesions involving the left and the right clivus, right Meckel's cave, and posterior margin of the right cavernous sinus. Findings also included pulmonary nodules and adenopathy supporting diagnosis of likely stage IV metastatic carcinoma. Further imaging revealed homogeneous enhancement of the uterus suggestive of diffusely infiltrative carcinoma; pathology results confirmed large cell neuroendocrine carcinoma of the uterine cervix (LCNEC) giving her a 1.5-month median survival range.

## 1. Introduction

Neuroendocrine tumors (NETs) of the cervix are rare entities and therefore their categorization continues to be refined. Both the World Health Organization (WHO) and the College of American Pathologist and the National Cancer Institute (CAPNCI) have added their input to this nomenclature. As there have only been a few cases reported, a consensus on histological diagnosis and appropriate treatment is ongoing. CAPNCI uses four general categories for neuroendocrine tumors of the uterine cervix, typical (classical) carcinoid tumor, atypical carcinoid tumor, large cell neuroendocrine carcinoma, and small (oat) cell carcinoma [1]. WHO uses similar categories with addition of grades 1 to 3 to further delineate aggressiveness of each type [2].

## 2. Case Report

**Figure 1:**
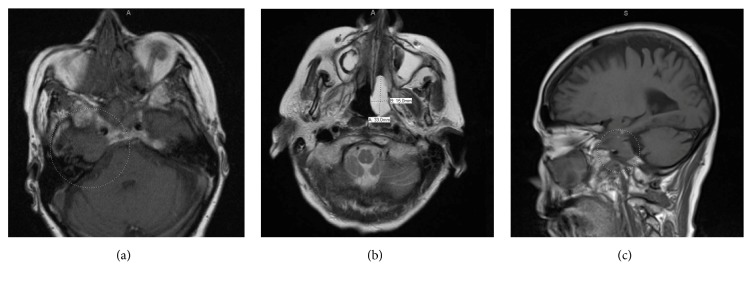
MRI brain: three abnormal bone marrow lesions (left clivus, right clivus, and occipital bone). The right clivus lesion appears to extend to the dura along the medial aspect of the middle cranial fossa which is likely the cause of cranial nerve sixth palsy. (a) Axial section at the level of the pons. (b) Axial section at the level of the medulla. (c) Sagittal section.

A 75-year-old Caucasian woman, gravida 5, para 3 with past medical history of hypertension, rheumatoid arthritis, and chronic obstructive pulmonary disease arrived to the emergency department looking for relief from dental pain. Neither initial exam nor maxillary plain film showed evidence of cause for facial pain, and she was admitted for further evaluation and pain control. On reevaluation, she was noted to have diplopia and facial droop, so MRI brain along with MRI/MRA of head and neck with and without contrast was ordered to rule out cerebrovascular accident (CVA). CVA was ruled out; however bone marrow lesions involving the left and the right clivus, right Meckel's cave, and posterior margin of the right cavernous sinus were noted (Figure 1).

**Figure 2:**
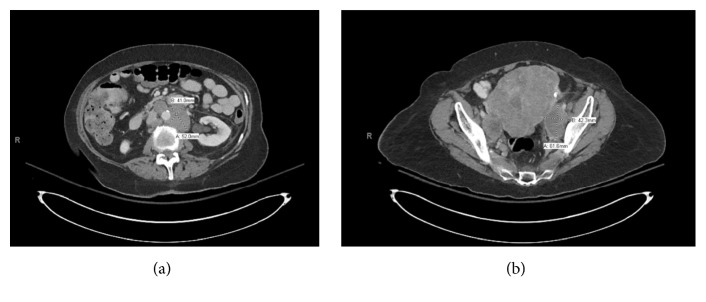
CT abdomen and pelvis: (a) bulky retroperitoneal adenopathy of the abdomen as well as bilateral iliac chain adenopathy. (b) Enlargement and nodularity of the uterus are seen, worrisome for diffusely infiltrative carcinoma.

CT scans of chest, abdomen, and pelvis with and without contrast were ordered to search for primary malignancy. These studies revealed homogeneous enhancement of the uterus concerning for diffusely infiltrative endometrial carcinoma with associated relatively bulky retroperitoneal adenopathy of the abdomen and bilateral iliac chain adenopathy. In addition, multiple pulmonary nodules were noted along with L sided neck, mediastinal, and right hilar adenopathy (Figure 2).

**Figure 3:**
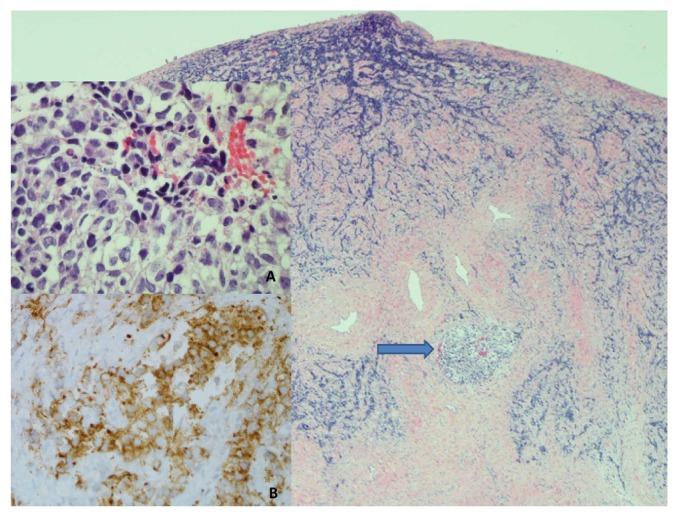
Cervical biopsy (40X): the infiltrative carcinoma is largely distorted by crush artifact. A preserved intravascular focus of carcinoma (arrow and inset A, 500X) is characterized by cells with variable clear to eosinophilic cytoplasm, round to oval nuclei, delicate chromatin, and punctate nucleoli. Scattered apoptotic cells are noted. The tumor cells are strongly immunoreactive for neuroendocrine markers synaptophysin (inset B, 400X).

Cervical biopsies were obtained disclosing LCNEC of the cervix (Figure 3). The tumor cells were immunoreactive for neuroendocrine markers synaptophysin and chromogranin. They were also immunoreactive for pancytokeratin and p16, the latter a surrogate marker for the presence of high-risk HPV often seen in these cervical carcinomas. The tumor cells lacked immunoreactivity for estrogen receptors and p63.

She was diagnosed with stage IV LCNEC with distant metastasis. This patient went on to receive palliative radiation to her brain for symptom control and was scheduled to see oncology as an outpatient to discuss treatments. However, due to decline in functional and mental status, she was no longer a candidate for chemotherapy and comfort care was pursued. She died 2 months after diagnosis.

## 3. Discussion

NETs are rare entities that arise from neurosecretory cells, which can be found in several organ systems throughout the body. These rare tumor types occur most commonly in the lungs and GI tract; however when it does occur in the genitourinary system, bladder seems to be the most common site [3]. Large cell carcinoma subtypes of NETs have very similar outcomes to their small cell carcinoma counterparts with median overall survival for stages 1, 2, 3, and 4 cancers being 19, 17, 3, and 1.5 months, respectively [1]. NETs typically present with either abnormal PAP smear or vaginal bleeding, which makes this case presentation rare as symptoms of her metastatic disease led to the investigation of the primary diagnosis.

Neuroendocrine differentiation is demonstrated with pan-neuroendocrine markers such as chromogranin A, synaptophysin, CD56, and neuron specific enolase. Although there can also be a variety of other peptides and hormones present, they have limited clinical significance [4]. These large cells, as their name suggests, are characterized by having large, prominent nucleoli, abundant cytoplasm and they undergo frequent mitoses [5]. There are tumor markers that have been identified as being expressed in these neuroendocrine cells, including TTF-1, which is a nuclear transcription factor regulator that is usually expressed in lung or thyroid tissue but can be seen in these tumors arising from other organs as well. Patients with International Federation of Gynecology and Obstetrics (FIGO) stage ≤ IIA, tumor size ≤ 4* *cm, depth of stromal invasion (DOI) ≤ 1/2, negative lymph node metastasis (LNM), and negative parametrial involvement (PMI) along with low expression of synaptophysin and chromogranin predict a better survival [6].

Treatment regimens for patients with LCNEC have long followed that of those with small cell neuroendocrine carcinoma, as this is a more common malignancy. Due to its rarity and poor prognostic features, LCNEC continues to be a difficult carcinoma to study and therefore treatment guidelines are nearly impossible to produce. Notably, although likely not surprising, tumor size seems to be an independent prognostic factor. In addition to this, whether disease is limited to the organ or has local or distal involvement plays a significant prognostic role as well [6].

Currently, the gold standard of treatment based on limited evidence has been radical surgical resection in conjunction with platinum-based chemotherapy and less likely radiotherapy [7, 8]. Not infrequently, neuroendocrine carcinomas of the cervix may have mixed histology with squamous cell carcinoma and/or adenocarcinoma elements. Some have suggested that adding radiation therapy to the adjuvant regimen is beneficial when confronted with tumors displaying mixed histology. This differentiation makes pathologic characterization even more important. Also, if the size of the tumor is small (that is <= 4cm), resection is likely just as efficient as resection and chemotherapy, with or without the addition of radiotherapy [6].

Even with this information, the overall recommendation is to still use adjuvant platinum-based chemotherapy in all cases with the addition of radiotherapy if squamous cell carcinoma or adenocarcinoma pathology is present [8]. The high recurrence rate and poor prognosis of this disease even in early stages make the need for a novel treatment imperative for improved outcomes for this disease. As the molecular and genetic understanding of NETs increases, creating targeted therapeutic options for these tumors, no matter the location within the body will become more of a reality. Plans for more research in this area are underway [4].
